# Psychometric evaluation of the Chinese CancerSupportSource^TM^-Caregiver among family caregivers to colorectal cancer patients using CTT and Rasch analyses

**DOI:** 10.1186/s12955-026-02507-x

**Published:** 2026-03-11

**Authors:** Rui Xia, Di Tian, Ying Zhou, Meihua Zhong, Yuxia Lin, Jinqiong He, Weilin Lin, Jin Li, Hui Li, Yuan Yang, Qiao Wang, Xueying Xiao, Xi Su

**Affiliations:** 1 School of Nursing, Guangzhou Medical University, No.1 Xinzao Road, Xinzao Town, Panyu District, Guangzhou, Guangdong 511436 China; 2 The Eighth Affiliated Hospital of Southern Medical University (The First People’s Hospital of Shunde), Foshan, Guangdong 528308 China; 3 The First Affiliated Hospital, Guangzhou Medical University, Guangzhou, Guangdong 510120 China; 4 Southern Medical University Hospital of Integrated Traditional Chinese and Western Medicine, Southern Medical University, No.13 Shi Liu Gang Road, Haizhu District, Guangzhou, Guangdong 510315 China; 5 Guangzhou Institute of Cancer Research, the Affiliated Cancer Hospital, Guangzhou Medical University, No.78, Hengzhi Gang Road, Yuexiu District, Guangzhou, Guangdong 510095 China

**Keywords:** Colorectal cancer, Classical test theory, Distress, Rasch analysis, Family caregivers, Psychometric properties

## Abstract

**Background:**

Family caregivers of colorectal cancer (CRC) patients often experience significant distress, yet no validated instrument exists in China to screen for this distress. This study aimed to translate the CancerSupportSource™-Caregiver (CSS-Caregiver) scale into Chinese and evaluate its psychometric properties among CRC family caregivers.

**Methods:**

A cross-sectional study was conducted with 340 family caregivers of CRC patients from three tertiary hospitals in Guangzhou. The Chinese CSS-Caregiver was translated and culturally adapted. Its psychometric properties were assessed using both Classical Test Theory and Rasch analysis. Reliability was evaluated via internal consistency and test-retest reliability. Validity was examined through confirmatory factor analysis (CFA), item-total correlations, and convergent validity. Measurement invariance was tested using multi-group CFA and Differential Item Functioning (DIF). The optimal cut-off score was determined via ROC analysis.

**Results:**

The Chinese CSS-Caregiver demonstrated excellent internal consistency (Cronbach’s α = 0.902) and test-retest reliability (ICC = 0.935). CFA supported the original five-factor structure with good model fit (χ²/df = 2.064, CFI = 0.956, RMSEA = 0.073). Rasch analysis indicated generally satisfactory item fit and person separation. The scale exhibited strict measurement invariance across sex, age, and residence, with negligible DIF. Strong convergent validity was demonstrated by correlations with the criterion measures (*r* = 0.81 ~ 0.85). An optimal cut-off score of 18.5 was established.

**Conclusions:**

The Chinese CSS-Caregiver is a valid and reliable instrument for assessing distress among family caregivers of CRC patients in China, facilitating early identification and timely psychosocial support.

**Supplementary Information:**

The online version contains supplementary material available at 10.1186/s12955-026-02507-x.

## Background

Family caregivers take on increased responsibility for the health care of individuals with cancer [1]. Population-based estimates have reported that family caregivers provide the majority of health care to cancer patients [2], including daily care, medical or nursing tasks (e.g., tube feeding, stoma care), and emotional support [3]. Providing care over a long period tends to cause physical exhaustion and a high care burden in caregivers, which leads to considerable psychological distress [4]. Psychological distress is a multidimensional construct referring to a state of emotional suffering characterized by symptoms such as anxiety, depressive mood, excessive worry, irritability, cognitive difficulties, and somatic complaints that arise when individuals perceive demands as exceeding their coping resources. It encompasses emotional, cognitive, and behavioral components and may impair daily functioning, interpersonal relationships, and overall quality of life. From a clinimetric perspective, psychological distress represents not only a continuous subjective experience but also a clinically actionable signal indicating the need for further assessment or intervention [5]. When unrecognized or unmanaged, distress can deplete caregivers’ psychological resources and affect their coping abilities, thereby exacerbating the negative impact of the caregiving burden and reducing their quality of life [6]. Moreover, distress can significantly impact caregivers’ daily functioning, and potentially have adverse effects on family relationships, social interactions, and work productivity [7]. Therefore, accurate and timely identification of psychological distress among caregivers is essential to facilitate appropriate supportive care and targeted interventions.

We focused specifically on caregivers of patients with colorectal cancer (CRC) because they play significant and changing roles from the time of diagnosis through treatment, well into survivorship or near end-of-life [8]. Within the Chinese sociocultural context, caregiving is predominantly family-centered, and family members are traditionally expected to assume primary responsibility for illness management and daily support. Such expectations may intensify emotional and psychological burden, particularly when formal caregiving resources are limited [9]. Moreover, caregiving for individuals with CRC often involves complex and long-term tasks, including stoma management, dietary modifications, monitoring of treatment-related side effects, and assistance with functional limitations [10]. These disease-specific demands may further increase caregivers’ psychological distress and unmet support needs. Given these disease-specific and culturally embedded challenges, the early identification of distress among CRC caregivers is a critical prerequisite for developing tailored psychological interventions and supportive care strategies. Despite CRC being the second most common cancer diagnosis in China [11], no validated measure allowing the identification of distress in family members of patients with CRC exists in China.

The Cancer Support Community has prioritized developing an instrument to objectively measure a caregiver’s level of distress. The Cancer Support Source ™-Caregiver (CSS-Caregiver), a web-based distress screening tool for cancer caregivers [12], has shown great potential in early identification of unmet caregiver needs and ensures timely referral and support. The CSS-Caregiver contains 18 items, plus one screening item assessing tobacco and substance use, designed to measure the caregiver’s current distress. The CSS-Caregiver is a multi-dimensional scale, and exploratory factor analysis (EFA) and confirmatory factor analysis (CFA) identified five factors: Patient Well-Being refers to the caregiver’s concern about the patient’s disease-related health conditions; Healthy Lifestyle emphasizes the caregiver’s concern about meeting their own health needs; Caregiving Tasks describes the caregiver’s concern for providing medical care to the patient; Finances involves the financial burden of caring for patients; and Emotional Well-Being includes items for anxiety and depression risk screening. The first subscale is commonly used to understand the caregiver’s concerns about the patient, whereas the latter four are commonly used to understand the caregiver’s concerns about themselves. In general, the scale considers the differences in individual emotions, covers all aspects of distress, is of an acceptable length, and has satisfactory psychometric properties.

Given that the CSS-Caregiver has recently been developed, only Zaleta and Ash-Lee’s original study has verified the psychometric properties of its English version. Consequently, there is little evidence of its psychometric properties in other cultures and languages. To ascertain the psychometric robustness of an instrument, its psychometric properties should be tested utilizing different statistical methods and across diverse populations [13]. Recent advances in scale validation recommend integrating classical test theory (CTT) and item response theory (IRT) methods [14]. CTT is a traditional and widely recognized framework for evaluating reliability and construct validity [15]. Concurrently, Rasch analysis, an IRT-based modeling approach, has increasingly been applied in the validation of psychological and health-related instruments [16]. Beyond conventional psychometric evaluation, Rasch analysis is also closely aligned with the principles of clinimetrics, an emerging scientific domain concerned with the development and validation of clinical measurement instruments that are not only statistically sound but also clinically meaningful and actionable. Clinimetrics emphasizes the practical utility of instruments in identifying clinically relevant thresholds, evaluating symptom severity, and supporting decision-making in real-world healthcare settings. Within this framework, Rasch modeling offers detailed item-level diagnostics, assessment of measurement invariance, and evaluation of scale functioning, thereby strengthening both measurement precision and clinical interpretability [17, 18]. Therefore, integrating CTT and Rasch analysis enables a comprehensive evaluation of both the psychometric properties and the clinimetric value of the CSS-Caregiver scale.

Accordingly, the present study aimed to translate and culturally adapt the CSS-Caregiver into Chinese and to conduct a comprehensive validation among caregivers of patients with CRC. By combining CTT and Rasch analyses, we sought to evaluate its reliability, structural validity, item-level functioning, measurement invariance, and clinical utility, including the identification of a clinically meaningful cut-off score for distress screening. The specific research questions were as follows:

1. What is the reliability of the Chinese CSS-Caregiver scale?
2. What is the validity of the Chinese CSS-Caregiver scale? Does the scale demonstrate satisfactory construct validity and an appropriate five-factor structure?
3. Does the scale have measurement invariance across age, sex, and residence groups?
4. What is the clinically meaningful cut-off score for identifying caregivers experiencing high levels of psychological distress?

## Methods

**Fig. 1:**
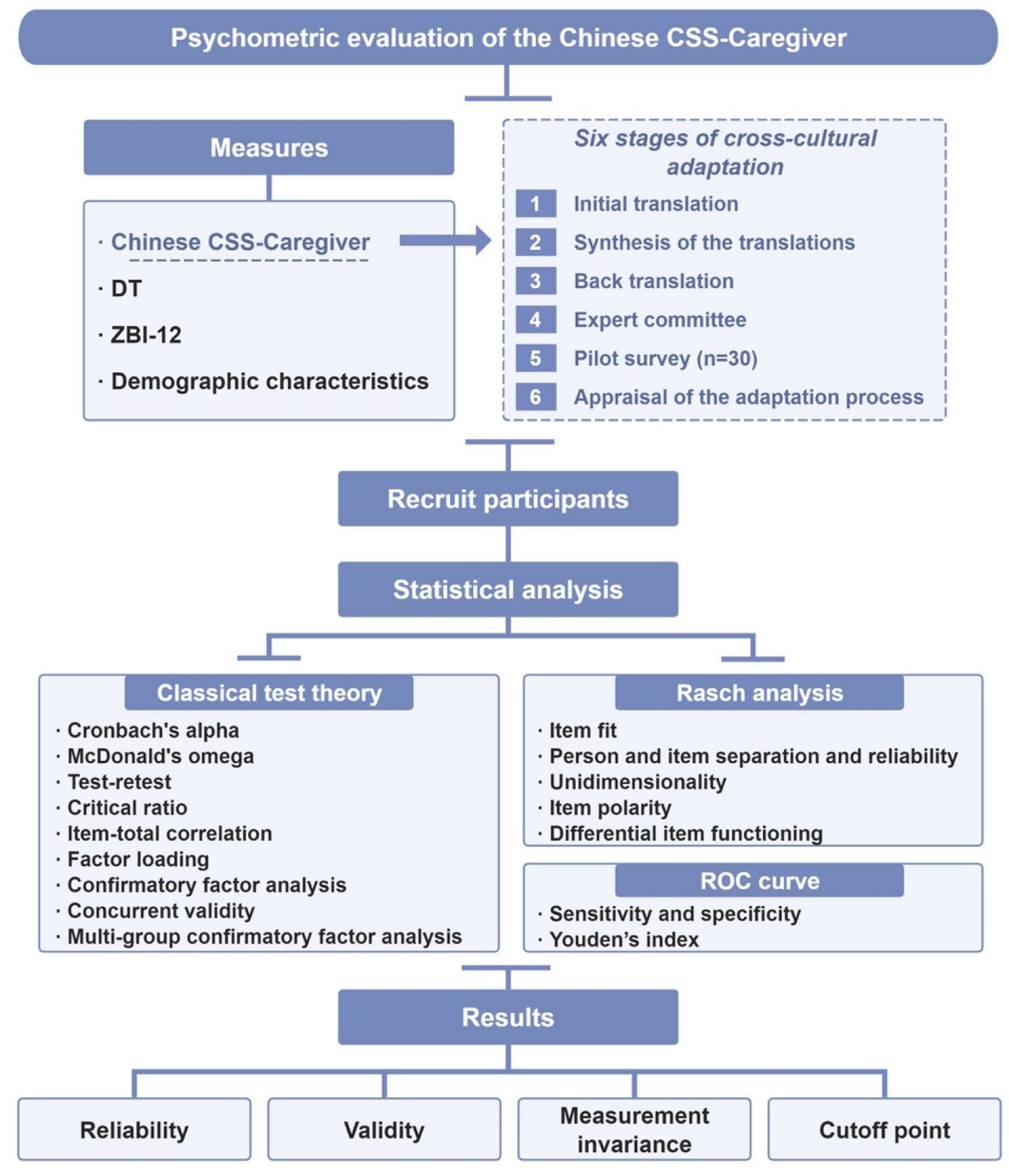
The design of the psychometric evaluation of the Chinese CSS-Caregiver

The current study design process is shown in Fig. 1.

### Measures

#### The Chinese version of CancerSupportSource ™-Caregiver (The Chinese CSS-Caregiver)

The CSS-Caregiver was translated into Chinese using forward translations and back translations according to recognized self-report methods [19]. We pursued a six-step process of translation as described below:

*Stage 1* Initial translations: two bilingual translators independently translated the original CSS-Caregiver into simplified Chinese.

*Stage 2* Synthesis of the translations: two translators and an observer synthesized the translations of the original questionnaire, creating a common version with a report detailing the process and resolutions. Consensus is key in resolving issues. The common version was then used for the next stage.

*Stage 3* Back translation: another two bilingual translators separately translated the common version into English.

*Stage 4* Expert committee: the nine-member expert panel (three clinical psychologists, one oncologist, three clinical oncology nurse specialists, one statistician, and one community management specialist) reviewed all the translations, reached a consensus on any discrepancies, and produced the prefinal version.

*Stage 5* Test of the prefinal version: 30 family caregivers to CRC patients at the hospital participated in the test. Each caregiver completed the questionnaire (prefinal version). We also conducted thorough interviews with the participants to ensure that all questions were unambiguous. Following this, the Chinese version of the CancerSupportSource ™-Caregiver (Chinese CSS-Caregiver) was finalized.

*Stage 6* Appraisal of the adaptation process: all the reports and forms were submitted to the original developers for appraisal of the adaptation process to complete the translation and cross-cultural adaptation.

The Chinese CSS-Caregiver aligns with the original version of the CSS-Caregiver in both item order and scoring method. Each item was measured on a 5-point Likert, as follows: 0 = Not at all, 1 = Slightly, 2 = Moderately, 3 = Seriously, 4 = Very Seriously. A total distress score was calculated as the sum of item ratings. A higher score indicates more severe distress in caregivers.

#### NCCN distress thermometer (DT)

The DT is a single-item tool consisting of an 11-point scale with responses ranging from 0 (no distress) to 10 (extreme distress). Participants rated their distress over the past week [20]. The Chinese version of the DT has demonstrated acceptable test–retest reliability (*r* = 0.80, *P* < 0.01), and a cutoff score of 4 is the most sensitive and specific [21], which is also suggested by NCCN distress management guidelines.

#### Zarit burden interview short form (ZBI-12)

The ZBI-12 (Cronbach’s alpha = 0.87) is a measure used to evaluate caregiver burden [22]. The scale consists of 12 items, each rated on a 5-point Likert scale ranging from 0 (never) to 4 (nearly always). The total score is calculated by summing the scores of all 12 items, with higher values indicating a higher burden.

#### Demographic characteristics of participants

Demographic data were collected on the caregivers (including age, sex, relationship to the patient, marital status, place of residence, education level, employment status, economic income, and months providing care), as well as clinical data on the patients (including diagnosis, cancer stage, type of cancer treatment, time since cancer diagnosis, recurrence/metastasis, presence of enterostomy, and chronic disease).

### Setting and participants

The psychometric evaluation of the Chinese CSS-Caregiver was conducted through a cross-sectional study in Guangzhou, China, by including gastroenterology departments of three tertiary hospitals in Guangzhou. The inclusion criteria for participants were as follows: (1) age ≥ 18 years old; (2) provided uncompensated care or assistance to a family member who had CRC; and (3) could understand and complete the questionnaire. The exclusion criteria were unstable systemic disease in family caregivers and providing care to more than one family member. The recommended sample size for CTT was 10 cases for each item [23], equating to 190 in this study, whereas the Rasch model required a sample size exceeding 200 [24]. Based on recommendations for CTT and Rasch analysis, the minimum required sample size for this study was estimated at 200 to ensure model stability. The final sample size exceeded this minimum requirement, and all samples were included in the CTT and Rasch analysis.

### Data collection

Investigators collected data for the questionnaires on-site and checked their completeness. The duration of the assessment for each respondent was approximately 10–15 min. The survey was anonymous, and the instructions for completing the questionnaire, along with the informed consent form, were printed on the first page. Each participant received a pack of tissues (approximately 0.6 pounds) in appreciation of their time and participation. Thirty caregivers were requested to leave their personal communication information to assess the reliability of the retest after 2 weeks. Data were collected from June to November 2024.

### Data analysis

The psychometric properties of the Chinese CSS-Caregiver were evaluated using both classical test theory (CTT) and Rasch analysis. The assessment followed established guidelines for scale development and validation (Table 1) [14, 25]. focusing on reliability, validity, and measurement invariance.

Reliability was assessed through internal consistency and temporal stability [26]. Within the CTT framework, we computed Cronbach’s alpha coefficient, McDonald’s omega coefficients, and test–retest intraclass correlation coefficients (ICC) from a subsample of 30 caregivers [27–29]. Item discrimination was evaluated using the critical ratio (CR > 3.0) [30]. In the Rasch model, item fit was examined via infit and outfit mean-square statistics (MNSQ) [27]. Person and item separation indices and reliability coefficients were analyzed to evaluate scale precision and stratification [15, 31].

Validity was assessed through multiple sources of evidence. Internal validity was examined using item-total correlations, factor loadings, confirmatory factor analysis (CFA), and unidimensionality tests [28]. Data suitability for factor analysis was confirmed with the Kaiser–Meyer–Olkin (KMO > 0.6) index and Bartlett’s test of sphericity (*P* < 0.05) [32]. CFA model fit was assessed using *χ*²/df, CFI, and RMSEA [28, 32]. Rasch principal component analysis of residuals evaluated unidimensionality [33], and point-measure correlations verified item alignment with the latent trait [27]. Convergent validity was established by correlating scale scores with the DT and ZBI-12 using Pearson’s coefficients [28].

Measurement invariance (MI) was tested via multi-group CFA [14] across age, sex, and residence. Sequential models (configural, metric, scalar) were compared using ΔCFI and ΔRMSEA. Differential item functioning (DIF) analysis was conducted at the item level; a DIF contrast ≥ 0.64 logits indicated significant bias between groups [34, 35].

The NCCN Distress Thermometer (cutoff ≥ 4) served as the criterion for ROC analysis. The optimal cutoff was identified using the Youden index [36, 37].

Descriptive statistics and CTT analyses were conducted using IBM SPSS 26.0 or AMOS 26.0. Rasch analyses, applying the Rating Scale Model (including DIF analysis), were performed using WINSTEPS, Version 4.5.4 (Winsteps^®^ Rasch Measurement, 2017).

**Table 1:** Summary of methods used in current study

Aspect of analysis	Analysis	Statistical Index	Reference Values
Reliability and precision assessment	CTT	Cronbach’s alpha and McDonald’s omega	> 0.8, good internal consistency
		Test-retest	Intraclass correlation coefficient (ICC) > 0.7
		Critical ratio (CR)	> 3.0
	Rasch	Item fit	Total mean square Infit and Outfit within 0.5 to 1.5
		Person separation and reliability	Person reliability > 0.8; Person separation index > 2.0
		Item separation and reliability	Item reliability > 0.8 Item separation index > 3.0
Validity assessment	CTT	Factor loading	> 0.5
		Item-total correlation	> 0.3
		Confirmatory factor analysis (CFA)	Degrees of freedom ratio (χ2/df) < 3.0, Comparative Fit Index (CFI) > 0.90, Root Mean Square Error of Approximation (RMSEA) < 0.08
		Concurrent validity	Pearson correlation coefficients between Chinese CSS-Caregiver with ZBI-12 and DT, person *r* values of 0.10, 0.30, and 0.50 to differentiate small, medium, and large effects
	Rasch	Unidimensionality	The unexplained variance in the first contrast should be less than 2.0 eigenvalue units
		Item polarity	Point measure correlation (PTMea Corr.) > 0
Measurement invariance	CTT	Multi-group confirmatory factor analysis	ΔCFI ≤ 0.02 and ΔRMSEA ≤ 0.03 for the model of measurement weights; ΔCFI ≤ 0.01 and ΔRMSEA ≤ 0.015 for the model of measurement intercepts
	Rasch	Differential item functioning (DIF)	A contrast of 0.64 logits or above indicates a significant DIF
Cutoff point	--	Area under the curve (AUC)	≥ 0.9
		Youden’s index	(Sensitivity + Specificity) – 1

## Results

### Characteristics of patients with CRC and caregivers

A total of 358 questionnaires were collected, of which 18 were excluded due to incomplete responses or suspicious uniformity, yielding 340 valid questionnaires (response rate: 94.97%). This final sample exceeded the minimum requirement of 200, thereby enhancing the robustness of the psychometric evaluation. All 340 participants were included in both CTT and Rasch analyses. Appendix 1 (Table A) summarizes descriptive statistics for sociodemographic and caregiving characteristics. Respondents were predominantly young or middle-aged adults. The gender distribution among the respondents was even. More than 50% reported a parent to be the care recipient, and 64.7% of participants reported fewer than 3 months of care provided. However, 51.5% reported more than 12 h of care provided daily.

### Reliability and precision

#### Cronbach’s alpha coefficients, McDonald’s omega coefficients, test-retest reliability, and critical ratio value

Cronbach’s alpha coefficients for the Chinese CSS-Caregiver subscales ranged from 0.790 to 0.908, McDonald’s omega coefficients ranged from 0.842 to 0.914, and the 2-week test-retest reliability coefficients ranged from 0.778 to 0.935 (Table 2). The CR values of all items on the scale were significant (*P* < 0.01) and above 3.0 (Table 3). These results confirmed that this psychometric test had high reliability.

#### Item fit, person and item separation, and reliability

The item fit, person and item separation, and reliability statistics for each item by subscale are displayed in Table 3. All 18 items, except for item 8 (Infit MNSQ = 1.54, Outfit MNSQ = 1.52) and item 14 (Outfit MNSQ = 1.52), showed acceptable-fit statistics from the initial analysis. The majority of the Rasch person reliabilities of the Chinese CSS-Caregiver were above 0.8 (with person separation index > 2.0). The person separation index of the Healthy Lifestyle subscale was 1.60, which was below 2.0. The Rasch item reliabilities in the Patient Well-Being, Caregiving Tasks, and Emotional Well-Being subscales were high: all approximately 1.0 (with an item separation index > 3.0).

**Table 2:** Psychometric properties of the Chinese CSS-Caregiver at scale level (*n* = 340)

	CTT					Rasch				
	Cronbach’s alpha	McDonald’s omega	Test-retest reliability	Correlation with DT	Correlation with ZBI-12	Person separation	Person reliability	Item separation	Item reliability	Unexplained variance in first contrast
The CSS-Caregiver 18+	0.938	0.942	0.876	0.852^**^	0.808^**^	3.32	0.92	8.91	0.99	2.9
Factor 1: Patient Well-Being	0.869	0.869	0.778	0.666^**^	0.605^**^	2.28	0.84	7.82	0.98	1.9
Factor 2: Healthy Lifestyle	0.840	0.842	0.935	0.543^**^	0.543^**^	1.60	0.72	0.59	0.26	1.5
Factor 3: Caregiving Tasks	0.884	0.914	0.866	0.727^**^	0.650^**^	2.43	0.86	4.88	0.96	1.9
Factor 4: Finances	0.790	--	0.843	0.661^**^	0.624^**^	2.20	0.83	1.38	0.65	0.0
Factor 5: Emotional Well-Being	0.908	0.910	0.839	0.791^**^	0.788^**^	2.42	0.85	7.36	0.98	1.7
**Quality criteria**	**> 0.7**	**> 0.7**	**> 0.7**	**> 0.5**	**> 0.5**	**> 2.0**	**> 0.8**	**> 3.0**	**> 0.8**	**< 2**

Abbreviations: ICCs = Intraclass Correlation Coefficients; CSS-CG‐18 + = CancerSupportSource^TM^‐Caregiver with 18‐item measure plus one screening item assessing tobacco and substance use. DT = Distress Thermometer; ZBI = Caregiver Burden Interview

^**^Correlation is significant at the 0.01 level (2-tailed)

**Table 3:** Psychometric properties of the Chinese CSS-Caregiver at item level (*n* = 340)

Item No.	CTT				Rasch		
	Factor loading	Item -Total correlation	Critical ratio	Cronbach’s α if the item will be deleted	Infit MNSQ	Outfit MNSQ	Item polarity (PTMea Corr.)
PW-1	0.721	0.723^**^	16.525^**^	0.935	0.88	0.89	0.86
PW-2	0.818	0.689^**^	12.500^**^	0.935	0.70	0.71	0.88
PW-3	0.761	0.624^**^	10.414^**^	0.937	1.17	1.08	0.83
PW-4	0.594	0.624^**^	12.149^**^	0.936	1.27	1.23	0.80
HL-1	0.750	0.598^**^	12.383^**^	0.937	1.05	1.01	0.88
HL-2	0.738	0.543^**^	9.306^**^	0.938	1.03	0.99	0.85
HL-3	0.774	0.620^**^	15.203^**^	0.936	0.90	0.89	0.88
CT-1	0.634	0.728^**^	17.985^**^	0.934	1.54	1.52	0.81
CT-2	0.771	0.718^**^	15.884^**^	0.935	1.03	1.01	0.89
CT-3	0.854	0.782^**^	22.955^**^	0.933	0.61	0.61	0.93
CT-4	0.821	0.791^**^	19.525^**^	0.933	0.80	0.81	0.91
FI-1	0.802	0.718^**^	18.972^**^	0.935	1.00	0.93	0.98
FI-2	0.842	0.716^**^	19.589^**^	0.935	1.00	0.93	0.98
EW-1	0.662	0.690^**^	15.975^**^	0.935	1.49	1.52	0.77
EW-2	0.745	0.831^**^	22.231^**^	0.932	0.69	0.67	0.88
EW-3	0.752	0.828^**^	21.346^**^	0.932	0.63	0.62	0.90
EW-4	0.684	0.763^**^	18.437^**^	0.934	0.81	0.79	0.88
EW-5	0.537	0.726^**^	14.699^**^	0.934	1.39	1.32	0.75
TS-1	--	0.235^**^	4.032^**^	0.942	--	--	--
**Cutoff value**	**> 0.5**	**> 0.3**	**> 3.0**	**--**	**0.5–1.5**	**0.5–1.5**	**> 0**

Abbreviations: PW=Patient Well-Being; HL=Healthy Lifestyle; CT= Caregiving Tasks; FI=Finances; EW=Emotional Well-Being; TS=Tobacco or Substance Use. CTT=Classic Test Theory

^**^Correlation is significant at the 0.01 level (2-tailed)

### Validity

#### Factor loading, item-total correlation, and concurrent validity

The factor loadings for the Chinese CSS-Caregiver ranged from 0.54 to 0.85. The item-total correlation coefficients ranged from 0.54 to 0.83 except for item TS-1 (Table 3). Pearson’s correlations of the Chinese CSS-Caregiver factors with validation measures confirmed good convergent validity. Table 2 summarizes the correlation between the total score for the Chinese CSS-Caregiver and factors, and the total score for the DT and ZBI-12 ranged from 0.54 to 0.85. Generally, greater total distress, as captured by the Chinese CSS-Caregiver, was associated with greater distress as measured by the DT (*r* = 0.85) and with greater caregiver burden (*r* = 0.81).

#### Confirmatory factor analysis

The KMO index was 0.902, and the chi-square value of Bartlett’s Test of Sphericity was 5002.796 (*P* < 0.001). Confirmatory factor analysis showed the goodness of fit indexes for the five-factor model to be *χ*^*2*^/*df* = 2.064, CFI = 0.956, and RMSEA = 0.073 (Table 4). The five-factor model outperformed the one-factor model in terms of goodness of fit, error size, and explanatory power, making it a more appropriate model to describe the structure of the Chinese CSS-Caregiver scale.

#### Unidimensionality and item polarity

Table 3 illustrates the results of the unidimensionality and item polarity analysis for the 18-item scale. The PCA of residuals demonstrated that the observed variance of each subscale ranged from 62.8% to 90.6%. The eigenvalue of the unexplained variance in the first contrast for PW, HL, CT, FI, and EW subscales was 1.9, 1.5, 1.9, 0.0, and 1.7, respectively. Interpretation of the result of the “Finances” subscale was less meaningful since it had only two items. As they explained above 40% of the variance, and the eigenvalue of unexplained variance in the first contrast was below 2.0, unidimensionality was supported for each subscale. Furthermore, all items demonstrated positive point-measure correlations, indicating that each item of the Chinese CSS-Caregiver aligned with the latent trait.

### Measurement invariance

Table 4 presents the fit indices for each measurement invariance model. Except for the one-factor model, all models demonstrated acceptable fit: *χ*^*2*^/*df* < 2.0, CFI > 0.90, RMSEA < 0.08. Furthermore, the analysis of metric invariance models and scalar invariance models indicated that the differences were trivial: ΔCFI _max_ < 0.01, ΔRMSEA _max_ < 0.015. The results demonstrated that the Chinese CSS-Caregiver could be considered fully invariant across sex, age, and residence. No item was found to exhibit DIF across sex and age levels (Appendix 2, Tables A and B). Item 14 (rural: − 0.54 vs. urban: 0.15, *p* < 0.001) of the Emotional Well-Being subscale exhibited DIF across residences: rural caregivers were more likely to endorse the item (Appendix 2, Table C).

**Table 4:** Structure and invariance across sex, age, and residence of the Chinese CSS-Caregiver

Full sample (*n* = 340)	χ^2^	df	χ^2^ /df	RMSEA	CFI	ΔRMSEA	ΔCFI	PASS
1-factor model of Chinese CSS-Caregiver	1927.599	135	14.279	0.198	0.638	--	--	No
5-factor model of Chinese CSS-Caregiver	249.704	121	2.064	0.073	0.956	--	--	Yes
Sex: Female (*n* = 152), male (*n* = 188)								
Model 1: Unconstrained	619.732	242	2.561	0.068	0.925	--	--	Yes
Model 2: Measurement weights	640.138	255	2.51	0.067	0.924	0.001	0.001	Yes
Model 3: Measurement intercepts	655.399	273	2.401	0.064	0.924	0.004	0.001	Yes
Age: Younger (*n* = 201), older (*n* = 139)								
Model 1: Unconstrained	582.039	242	2.405	0.065	0.933	--	--	Yes
Model 2: Measurement weights	593.865	255	2.329	0.063	0.933	0.002	0.000	Yes
Model 3: Measurement intercepts	614.907	273	2.252	0.061	0.932	0.004	0.001	Yes
Residence: Rural (*n* = 262), urban (*n* = 78)								
Model 1: Unconstrained	674.357	242	2.787	0.073	0.916	--	--	Yes
Model 2: Measurement weights	688.104	255	2.698	0.071	0.916	0.002	0.000	Yes
Model 3: Measurement intercepts	709.112	273	2.597	0.069	0.915	0.004	0.001	Yes

Model 1 unconstrained = configural invariance. Model 2 measurement weights = metric invariance. Model 3 measurement intercepts = scalar invariance

### The cut-off point

The Chinese CSS-Caregiver score differed significantly between distressed individuals and non-distressed individuals (*P* < 0.05) with acceptable discriminative accuracy (AUC = 0.92, 95%CI 0.88–0.96). The Youden’s index had a maximum value of 0.713, corresponding to the coordinates (0.854, 0.141), which meant the sensitivity was 0.854 and the specificity was 0.859. The specific stage values corresponding to sensitivity and specificity can be seen in Appendix 3 (Table A). Thus, the optimal cut-off point for the Chinese CSS-Caregiver was 18.5 points.

## Discussion

This study adhered to the Beaton guideline for the translation of the CSS-Caregiver into Chinese [19]. By employing advanced methods of classical test theory and Rasch analysis, this study conducted a comprehensive evaluation of the psychometric properties of the Chinese CSS-Caregiver.

### The reliability of the scale was satisfactory through multiple validations

Both the Cronbach’s alpha and McDonald’s omega coefficients of the scale indicated a satisfactory internal consistency. The scale developers cited a study limitation as “the time interval for test-retest reliability was relatively short” [12]. We took into account the original author’s recommendations and the literature on similar studies when designing our study; therefore, we set the test-retest reliability interval at 2 weeks [32]. The test-retest reliability values of the scale and of each subscale were acceptable, indicating that the scale possessed a certain cross-time stability in measuring the existential distress of the caregivers of patients with CRC. In the Rasch model, reliability analyses indicated good person separation for the majority of subscales, suggesting that the Chinese CSS-Caregiver subscales were sensitive and precise enough to classify respondents into different strata of distress symptoms [14]. However, the item reliability of the Finance subscale did not meet the criterion of 0.80. We reviewed the DIF of the items of the Finance subscale, and both items were found to exhibit DIF across age levels, which may have influenced the reliability [38]. At the item level, all items showed satisfactory reliability and precision in CTT. However, in Rasch analysis, the infit and outfit MNSQ of item CT-1 (Providing transportation to treatment and appointments) and item EW-1 (Changes or disruptions in work, school, or home life) exceeded 1.5, which indicated underfitting. Linacre suggested that an MNSQ value between 1.5 and 2.0 was unproductive for the construction of a measurement tool, but did not degrade the scale [39]. The underfitting of these items may be attributed to their strong dependence on external factors—CT-1 is influenced by family support networks and transportation availability, while EW-1 may be interpreted differently depending on employment type and urban-rural context. Such contextual variations can reduce the consistent contribution of these items to the same latent trait. Future validation should include cognitive interviews to clarify item comprehension and consider wording refinements or item additions to improve subscale performance [40]. Overall, these findings responded adequately to Question 1: the reliability and precision of the Chinese CSS-Caregiver were satisfactory at both scale and item levels.

### The validity of the scale was good, and the five-factor structure was reasonable

In the structure validity analysis, the focus was to investigate a more appropriate factorial structure of the CSS-Caregiver, with a particular emphasis on improving and promoting this Chinese CSS-Caregiver adaptation. The dimensionality analysis suggested that the Chinese CSS-Caregiver scale had a multidimensional structure because the unexplained variance in the first contrast was greater than 2.0 in the PCA. Furthermore, as the unexplained variance in the first contrast of five subscales was below 2.0, unidimensionality was supported for each subscale. CFA suggested that Zaleta’s five-factor model should be the final measurement model, justifying the use of five unidimensional subscale scores to characterize distress. The five-factor model is theoretically aligned with the multidimensional nature of distress [41]. We are confident that the structure of the five factors of the Chinese CSS-Caregiver was reasonable and optimal. Criterion validation involved comparing the new instrument with established measures of the same construct, a process often referred to as criterion validity [26]. In this study, the Chinese DT and ZBI-12 were utilized as the criterion, and personal correlations of Chinese CSS-Caregiver factors with validation measures confirmed good convergent validity. These results are consistent with previous literature that corroborates a close association between care burden and distress [6, 12]. These findings responded to Question 2: the scale had good internal and external validity, and the five-factor structure was reasonable.

### The measurement invariance and stability of the scale were adequate

Measurement invariance was evaluated to examine the psychometric equivalence of the Chinese CSS-Caregiver across sociodemographic variables among CRC caregivers. Our findings affirmed measurement invariance within the five-factor structure, demonstrating equal factor loadings and latent means across both sex and age groups. This consistency aligned with prior research that has established measurement invariance by age and sex subgroups [14, 28]. It indicated that the construct of the Chinese CSS-Caregiver was uniformly comprehended by participants across different sexes and age subgroups, with no discernible systematic bias linked to the measurement factors. In addition, the DIF further revealed items that had statistical significance across sex, age, and residence. No item was found to exhibit DIF across sex and age levels. Only item 14 (Changes or disruptions in work, school, or home life) of the Emotional Well-Being subscale showed DIF between caregivers from different residential areas (urban and rural). Specifically, rural caregivers were more likely to endorse the item than their urban counterparts. This difference may reflect the different experiences or expressions of emotional health among caregivers in various residential settings. This finding suggested that when interpreting the results of this scale, the impact of residence should be taken into account, and further cultural adaptation or revision may be needed to ensure the fairness and accuracy of the scale in different residential contexts [14]. Despite the presence of DIF, the Chinese CSS-Caregiver still demonstrated a high degree of measurement invariance in general. Overall, in response to Question 3, the Chinese CSS-Caregiver scale had a high degree of measurement invariance in cross-group studies.

### The usability of the scale was enhanced by finding the optimal cut-off point

We used the Youden index to select the optimal cutoff point, which is by far the most common method used in accuracy studies of distress screening measures [21, 42]. The Chinese CSS-Caregiver effectively distinguished between distressed and non-distressed individuals, with high discriminative accuracy (AUC = 0.92). The optimal cut-off score of 18.5 balanced sensitivity (0.854) and specificity (0.859), maximizing the Youden index (0.713), establishing a practical threshold for identifying caregivers at risk of clinically significant distress. Based on this threshold, the scale can be integrated into routine outpatient or post-discharge assessments, administered by nurses or social workers in approximately 10 min. Scores ≥ 18.5 should prompt further evaluation using the Distress Thermometer or clinical interview, with referral to psychological services as indicated. Implementation requires attention to several considerations. Frontline staff need brief training on scale administration and the item-level limitations identified—particularly that certain items (e.g., CT-1, EW-1) may be interpreted differently across cultural or socioeconomic contexts. The two-item Finances subscale should be interpreted cautiously. Future research should evaluate operational outcomes including staff acceptability, referral rates, and intervention effectiveness. This dual reporting of psychometric properties and clinical threshold supports evidence-based screening while acknowledging practical challenges, enhancing the scale’s applicability in busy clinical settings.

### Clinical Implications and limitation

From both psychometric and clinimetric perspectives, this study confirms that the Chinese CSS-Caregiver possesses robust reliability and structural validity, while also establishing a clinically meaningful cut-off score (≥ 18.5) via ROC analysis. This dual reporting enables direct translation of scale results into clinical decision-making, such as outpatient screening and targeted referral pathways. By identifying and quantifying caregiver distress, the scale addresses a multidimensional issue spanning individual, family, and societal levels. Its use may indirectly improve patient recovery outcomes and provide empirical data to inform health policy and resource allocation for caregiver support. However, several limitations warrant consideration. First, the sample was predominantly recruited from tertiary hospitals in South China, limiting generalizability to other regions or community settings. Second, Rasch analysis revealed underfitting for items CT-1 and EW-1, suggesting semantic or contextual variations may affect their performance. Future research should validate the scale in more diverse samples, conduct cognitive interviews to explore item comprehension, and consider minor wording refinements or item additions to enhance subscale stability. Clinicians should interpret subscale-specific scores with appropriate caution when applying the scale in practice.

## Conclusion

This study translated the CSS-Caregiver into Chinese and comprehensively evaluated its psychometric properties among family caregivers of CRC patients using both CTT and Rasch analysis. The Chinese CSS-Caregiver demonstrated robust reliability, structural validity, and measurement invariance across key demographic subgroups. The established cut-off score of 18.5 provides a clinically useful threshold for identifying caregivers at risk of significant distress, facilitating early screening and timely psychosocial support in busy clinical settings. While further validation in more diverse populations is warranted, the current findings support the Chinese CSS-Caregiver as a reliable and valid instrument for both research and clinical practice.

## Supplementary Information

Below is the link to the electronic supplementary material.

Supplementary Material 1

Supplementary Material 2

Supplementary Material 3

## Data Availability

The datasets generated and analysed during the current study are not publicly available due to privacy and confidentiality reasons, but are available from the corresponding author on reasonable request.

## Abbreviations

AUC: Area Under the Curve
CFA: Confirmatory Factor Analysis
CRC: Colorectal Cancer
CR: Critical Ratio
CTT: Classical Test Theory
DIF: Differential Item Functioning
DT: Distress Thermometer
ICC: Intraclass Correlation Coefficient
IRT: Item Response Theory
KMO: Kaiser-Meyer-Olkin
MNSQ: Mean-Square
PCA: Principal Component Analysis
ROC: Receiver Operating Characteristic
RMSEA: Root Mean Square Error of Approximation
CFI: Comparative Fit Index
