# Supplementary material for: Psychometric evaluation of the Chinese CancerSupportSourceTM-Caregiver among family caregivers to colorectal cancer patients using CTT and Rasch analyses
Source: Health Qual Life Outcomes. 2026 Mar 11;24:48. doi: 10.1186/s12955-026-02507-x (PMC13088487; doi:10.1186/s12955-026-02507-x)
Supplement: Supplementary file 1 — Supplementary Material 1 [file 12955_2026_2507_MOESM1_ESM.docx]

**Appendix 1**

Table A. Demographic characteristics of CRC cancer patients and family caregivers (*n*=340)

| Characteristics | Family Caregivers | | Colorectal Cancer Patients | |
| --- | --- | --- | --- | --- |
|  | *n* | % | *n* | % |
| Relationship to patient |  |  |  |  |
| Spouse | 97 | 28.5 |  |  |
| Child | 185 | 54.4 |  |  |
| Parents | 15 | 4.4 |  |  |
| Relatives | 43 | 12.6 |  |  |
| Age |  |  |  |  |
| Younger(18-44 ) | 201 | 59.1 | 59 | 17.4 |
| Older (≥ 45) | 139 | 40.9 | 281 | 82.6 |
| Sex |  |  |  |  |
| Female | 152 | 44.7 | 138 | 59.4 |
| Male | 188 | 55.3 | 202 | 40.6 |
| Marital status |  |  |  |  |
| Unmarried | 62 | 18.2 | 10 | 2.9 |
| Married | 269 | 79.1 | 306 | 90.0 |
| Divorced or widowed | 9 | 2.6 | 24 | 7.1 |
| Place of residence |  |  |  |  |
| Rural | 262 | 77.1 | 257 | 75.6 |
| Urban | 78 | 22.9 | 83 | 24.4 |
| Education level |  |  |  |  |
| Elementary school and below | 32 | 9.4 | 148 | 43.5 |
| Junior high school | 96 | 28.2 | 90 | 26.5 |
| Senior high school | 69 | 20.2 | 54 | 15.9 |
| University and above | 143 | 42.1 | 48 | 14.1 |
| Employment status |  |  |  |  |
| Full time jobs | 121 | 35.6 |  |  |
| Part time jobs | 132 | 38.8 |  |  |
| No jobs | 53 | 15.6 |  |  |
| Retire | 34 | 10.0 |  |  |
| Monthly household income (yuan) |  |  |  |  |
| Below 3000 | 62 | 18.2 |  |  |
| 3000-4999 | 101 | 29.7 |  |  |
| 5000-7999 | 69 | 20.3 |  |  |
| Above 8000 | 65 | 19.1 |  |  |
| Prefer not to share | 43 | 12.7 |  |  |
| Co-caregivers |  |  |  |  |
| No | 269 | 79.1 |  |  |
| Yes | 71 | 20.9 |  |  |
| Months providing care |  |  |  |  |
| Below 3 months | 220 | 64.7 |  |  |
| 3-6 months | 30 | 8.8 |  |  |
| Above 6months | 90 | 26.5 |  |  |
| Hours of care provided daily |  |  |  |  |
| Below 6 hours | 85 | 25 |  |  |
| 6-12 hours | 80 | 23.5 |  |  |
| Above 12 hours | 175 | 51.5 |  |  |
| Diagnosis |  |  |  |  |
| Colon cancer |  |  | 199 | 58.5 |
| Rectal Cancer |  |  | 141 | 41.5 |
| Cancer stage |  |  |  |  |
| Ⅰ |  |  | 3 | 0.9 |
| Ⅱ |  |  | 21 | 6.2 |
| Ⅲ |  |  | 155 | 45.6 |
| Ⅳ |  |  | 161 | 47.4 |
| Type of cancer treatment |  |  |  |  |
| Only surgery |  |  | 173 | 50.9 |
| Only Chemotherapy |  |  | 20 | 5.9 |
| Combination therapy |  |  | 147 | 43.2 |
| Time since cancer diagnosis |  |  |  |  |
| Below 3 months |  |  | 119 | 35.0 |
| 3-12 months |  |  | 139 | 40.9 |
| 13-36 months |  |  | 40 | 11.8 |
| Above 36 months |  |  | 42 | 12.4 |
| Recurrence/metastasis |  |  |  |  |
| No |  |  | 295 | 86.8 |
| Yes |  |  | 45 | 13..2 |
| Stoma |  |  |  |  |
| No |  |  | 271 | 79.7 |
| Yes |  |  | 69 | 20.3 |
