# Supplementary material for: Psychometric evaluation of the Chinese CancerSupportSourceTM-Caregiver among family caregivers to colorectal cancer patients using CTT and Rasch analyses
Source: Health Qual Life Outcomes. 2026 Mar 11;24:48. doi: 10.1186/s12955-026-02507-x (PMC13088487; doi:10.1186/s12955-026-02507-x)
Supplement: Supplementary file 2 — Supplementary Material 2 [file 12955_2026_2507_MOESM2_ESM.docx]

**Appendix 2**

Table A. Differential item functioning of the Chinese CSS-Caregiver across sex subgroups

| Item  No. | DIF contrast  logit | Mantel–Haenszel  chi-square *P* | | DIF Measure  Female Male | |
| --- | --- | --- | --- | --- | --- |
| 01 | 0.11 | 0.3201 | 0.5715 | -0.60 | -0.72 |
| 02 | -0.07 | 2.1710 | 0.1406 | -0.27 | -0.20 |
| 03 | -0.41 | 9.6145 | 0.0019 | 0.11 | 0.52 |
| 04 | 0.20 | 0.0576 | 0.8103 | -0.64 | -0.84 |
| 05 | 0.20 | 0.2674 | 0.6051 | 1.19 | 0.99 |
| 06 | 0.30 | 0.0026 | 0.9596 | 1.14 | 0.83 |
| 07 | 0.51 | 1.5720 | 0.2099 | 1.22 | 0.71 |
| 08 | -0.18 | 0.6985 | 0.4033 | 0.49 | 0.67 |
| 09 | -0.31 | 3.8095 | 0.0510 | -0.17 | 0.15 |
| 10 | -0.23 | 0.7485 | 0.3869 | -0.12 | 0.11 |
| 11 | -0.28 | 1.1298 | 0.2878 | -0.12 | 0.16 |
| 12 | 0.55 | 17.1724 | <0.001 | -0.46 | -1.01 |
| 13 | 0.54 | 16.9761 | <0.001 | -0.53 | -1.08 |
| 14 | 0.13 | 3.7780 | 0.0519 | -0.31 | -0.44 |
| 15 | -0.18 | 0.2316 | 0.6303 | -0.19 | 0.00 |
| 16 | -0.25 | 0.3460 | 0.5564 | -0.42 | -0.17 |
| 17 | -0.34 | 1.0083 | 0.3153 | -0.78 | -0.44 |
| 18 | -0.28 | 1.9210 | 0.1657 | -0.55 | 0.83 |

Abbreviation: DIF = differential item functioning

DIF across sex subgroups (female, N =152 and male, N = 188)

Table B. Differential item functioning of the Chinese CSS-Caregiver across age subgroups

| Item  No. | DIF contrast  logit | Mantel–Haenszel  chi-square *P* | | DIF Measure  Younger Older | |
| --- | --- | --- | --- | --- | --- |
| 01 | 0.22 | 6.6675 | 0.0098 | -0.58 | -0.79 |
| 02 | 0.06 | 0.2291 | 0.6322 | -0.21 | -0.27 |
| 03 | -0.26 | 1.4345 | 0.2310 | 0.21 | 0.48 |
| 04 | 0.12 | 1.6267 | 0.2022 | -0.70 | -0.83 |
| 05 | 0.39 | 10.4143 | 0.0013 | 1.26 | 0.87 |
| 06 | -0.13 | 0. 3192 | 0.5721 | 0.93 | 1.05 |
| 07 | 0.00 | 1.9238 | 0.1654 | 0.94 | 0.94 |
| 08 | -0.27 | 2.7925 | 0.0947 | 0.47 | 0.74 |
| 09 | -0.43 | 8.1326 | 0.0043 | -0.18 | 0.26 |
| 10 | -0.09 | 0.0830 | 0.7733 | -0.04 | 0.05 |
| 11 | 0.10 | 0.7183 | 0.3967 | 0.06 | -0.03 |
| 12 | -0.03 | 1.6701 | 0.1962 | -0.77 | -0.74 |
| 13 | 0.19 | 0.0597 | 0.8070 | -0.76 | -0.95 |
| 14 | -0.23 | 2.1652 | 0.1412 | -0.48 | -0.24 |
| 15 | -0.09 | 4. 4093 | 0.0357 | -0.13 | -0.03 |
| 16 | 0.20 | 0.0861 | 0.7692 | -0.21 | -0.40 |
| 17 | 0.05 | 0.1060 | .7448 | -0.58 | -0.63 |
| 18 | 0.24 | 0.7960 | 0.3723 | 0.80 | 0.55 |

Abbreviation: DIF = differential item functioning

DIF across age subgroups (Younger, N =201 and older, N =139)

Table C. Differential item functioning of the Chinese CSS-Caregiver across residence subgroups

| Item  No. | DIF contrast  logit | Mantel–Haenszel  chi-square *P* | | DIF Measure  Rural Urban | |
| --- | --- | --- | --- | --- | --- |
| 01 | -0.16 | 0.0120 | 0.9128 | -0.70 | -0.54 |
| 02 | 0.04 | 0.7448 | 0.3881 | -0.23 | -0.27 |
| 03 | 0.19 | 2.4403 | 0.1183 | 0.36 | 0.18 |
| 04 | -0.04 | 0.5188 | 0.4713 | -0.75 | -0.71 |
| 05 | 0.58 | 2.4747 | 0.1157 | 1.24 | 0.65 |
| 06 | 0.60 | 4.4010 | 0.0359 | 1.13 | 0.53 |
| 07 | 0.49 | 2.7866 | 0.0951 | 1.07 | 0.58 |
| 08 | 0.13 | 0.3777 | 0.5388 | 0.62 | 0.49 |
| 09 | 0.36 | 2.2361 | 0.1348 | 0.09 | -0.27 |
| 10 | 0.41 | 10.9234 | 0.0009 | 0.10 | -0.31 |
| 11 | 0.21 | 2.4857 | 0.1149 | 0.07 | -0.13 |
| 12 | -0.52 | 6.8976 | 0.0086 | -0.89 | -0.37 |
| 13 | -0.56 | 5.8669 | 0.0154 | -0.97 | -0.41 |
| 14 | **-0.70** | 15.1628 | **0.0001** | -0.54 | 0.15 |
| 15 | -0.43 | 15.9017 | 0.0001 | -0.19 | 0.24 |
| 16 | -0.10 | 0.0066 | 0.9351 | -0.31 | -0.21 |
| 17 | 0.23 | 0.7913 | 0.3737 | -0.54 | -0.77 |
| 18 | -0.31 | 5.9793 | 0.0145 | 0.62 | 0.94 |

Abbreviation: DIF = differential item functioning

DIF across residence subgroups (Rural, N =262 and urban, N =78)

DIF contrast logit values (in bold) for item 14 was higher than or equal to 0.64.
