# Supplementary material for: Psychometric evaluation of the Chinese CancerSupportSourceTM-Caregiver among family caregivers to colorectal cancer patients using CTT and Rasch analyses
Source: Health Qual Life Outcomes. 2026 Mar 11;24:48. doi: 10.1186/s12955-026-02507-x (PMC13088487; doi:10.1186/s12955-026-02507-x)
Supplement: Supplementary file 3 — Supplementary Material 3 [file 12955_2026_2507_MOESM3_ESM.docx]

**Appendix 3**

Table A. Coordinates of the Curve

| **Test Result Variable(s)** | **Sensitivity** | **1 - Specificity** | **Specificity** | **Youden’s index** |
| --- | --- | --- | --- | --- |
| 0 | 1 | 1 | 0 | 0 |
| 1.5 | 1 | 0.972 | 0.028 | 0.028 |
| 2.5 | 1 | 0.93 | 0.07 | 0.07 |
| 3.5 | 1 | 0.901 | 0.099 | 0.099 |
| 4.5 | 1 | 0.831 | 0.169 | 0.169 |
| 5.5 | 0.994 | 0.746 | 0.254 | 0.248 |
| 6.5 | 0.994 | 0.662 | 0.338 | 0.332 |
| 7.5 | 0.988 | 0.606 | 0.394 | 0.382 |
| 9 | 0.976 | 0.592 | 0.408 | 0.384 |
| 10.5 | 0.976 | 0.549 | 0.451 | 0.427 |
| 11.5 | 0.97 | 0.437 | 0.563 | 0.533 |
| 12.5 | 0.963 | 0.366 | 0.634 | 0.597 |
| 13.5 | 0.963 | 0.338 | 0.662 | 0.625 |
| 14.5 | 0.939 | 0.31 | 0.69 | 0.629 |
| 15.5 | 0.927 | 0.268 | 0.732 | 0.659 |
| 16.5 | 0.902 | 0.211 | 0.789 | 0.691 |
| 17.5 | 0.866 | 0.155 | 0.845 | 0.711 |
| **18.5** | **0.854** | **0.141** | **0.859** | **0.713** |
| 19.5 | 0.817 | 0.127 | 0.873 | 0.69 |
| 20.5 | 0.78 | 0.085 | 0.915 | 0.695 |
| 21.5 | 0.744 | 0.085 | 0.915 | 0.659 |
| 22.5 | 0.707 | 0.056 | 0.944 | 0.651 |
| 23.5 | 0.659 | 0.056 | 0.944 | 0.603 |
| 24.5 | 0.616 | 0.056 | 0.944 | 0.56 |
| 25.5 | 0.573 | 0.028 | 0.972 | 0.545 |
| 26.5 | 0.53 | 0.028 | 0.972 | 0.502 |
| 27.5 | 0.488 | 0.028 | 0.972 | 0.46 |
| 28.5 | 0.47 | 0.028 | 0.972 | 0.442 |
| 29.5 | 0.445 | 0.014 | 0.986 | 0.431 |
| 30.5 | 0.427 | 0.014 | 0.986 | 0.413 |
| 31.5 | 0.402 | 0.014 | 0.986 | 0.388 |
| 32.5 | 0.378 | 0.014 | 0.986 | 0.364 |
| 33.5 | 0.341 | 0.014 | 0.986 | 0.327 |
| 34.5 | 0.317 | 0.014 | 0.986 | 0.303 |
| 35.5 | 0.305 | 0.014 | 0.986 | 0.291 |
| 36.5 | 0.287 | 0.014 | 0.986 | 0.273 |
| 37.5 | 0.274 | 0.014 | 0.986 | 0.26 |
| 38.5 | 0.262 | 0 | 1 | 0.262 |
| 39.5 | 0.25 | 0 | 1 | 0.25 |
| 40.5 | 0.22 | 0 | 1 | 0.22 |
| 41.5 | 0.195 | 0 | 1 | 0.195 |
| 42.5 | 0.177 | 0 | 1 | 0.177 |
| 43.5 | 0.165 | 0 | 1 | 0.165 |
| 45 | 0.152 | 0 | 1 | 0.152 |
| 46.5 | 0.14 | 0 | 1 | 0.14 |
| 47.5 | 0.122 | 0 | 1 | 0.122 |
| 48.5 | 0.11 | 0 | 1 | 0.11 |
| 50 | 0.098 | 0 | 1 | 0.098 |
| 51.5 | 0.091 | 0 | 1 | 0.091 |
| 53.5 | 0.073 | 0 | 1 | 0.073 |
| 55.5 | 0.043 | 0 | 1 | 0.043 |
| 57.5 | 0.03 | 0 | 1 | 0.03 |
| 60 | 0.018 | 0 | 1 | 0.018 |
| 61.5 | 0.012 | 0 | 1 | 0.012 |
| 64 | 0.006 | 0 | 1 | 0.006 |
| 67 | 0 | 0 | 1 | 0 |
